# Robust Statistics for GNSS Positioning under Harsh Conditions: A Useful Tool?

**DOI:** 10.3390/s19245402

**Published:** 2019-12-07

**Authors:** Daniel Medina, Haoqing Li, Jordi Vilà-Valls, Pau Closas

**Affiliations:** 1 Institute of Communications and Navigation, German Aerospace Center (DLR), 17235 Neustrelitz, Germany; 2 Electrical and Computer Engineering Dept., Northeastern University, Boston, MA 02115, USA; li.haoq@husky.neu.edu (H.L.); closas@ece.neu.edu (P.C.); 3 Institut Supérieur de l’Aéronautique et de l’Espace (ISAE-SUPAERO), University of Toulouse, 31055 Toulouse, France; jordi.vila-valls@isae-supaero.fr

**Keywords:** robust statistics, global navigation satellite systems (GNSS), multipath, single point positioning (SPP)

## Abstract

Navigation problems are generally solved applying least-squares (LS) adjustments. Techniques based on LS can be shown to perform optimally when the system noise is Gaussian distributed and the parametric model is accurately known. Unfortunately, real world problems usually contain unexpectedly large errors, so-called outliers, that violate the noise model assumption, leading to a spoiled solution estimation. In this work, the framework of robust statistics is explored to provide robust solutions to the global navigation satellite systems (GNSS) single point positioning (SPP) problem. Considering that GNSS observables may be contaminated by erroneous measurements, we survey the most popular approaches for robust regression (M-, S-, and MM-estimators) and how they can be adapted into a general methodology for robust GNSS positioning. We provide both theoretical insights and validation over experimental datasets, which serves in discussing the robust methods in detail.

## 1. Introduction

Global navigation satellite systems (GNSS) play a fundamental role on prospective applications of intelligent transportation systems (ITS), as the main source of positioning information [1]. Besides, GNSS provides timing synchronization to critical applications such as the power grid or the stock market [2]. However, GNSS performance can be easily degraded by natural phenomena and signal reflection. Navigation in urban scenarios results particularly challenging due to the presence of severe multipath effects, inducing large errors in the observed pseudorange measurements. Most positioning techniques are based on maximum likelihood (ML) estimation, since it provides optimal solutions under the assumption of Gaussian distributed observation noise. Although this assumption is generally fulfilled for nominal GNSS open-sky conditions, positioning on signal-degraded scenarios constitutes a challenge for ML estimators such as the least-squares (LS) [3].

Thus, the GNSS community has devoted great efforts towards the development of resilient navigation solutions [4]. One of the most popular approaches is based on solution separation—also known as consistency-checking—where a statistical test is applied to the estimated residuals to verify whether the Gaussian assumption is fulfilled. Otherwise, combinations of subsets excluding one observation are computed and the statistical test applied again. This procedure is repeated until a fault-free subset is found. Advanced receiver autonomous integrity monitoring (ARAIM) is possibly the most well-known representative of the solution separation approach, becoming the de facto navigation method for vertical guidance in the aviation domain [5,6]. Numerous other works have adapted consistency-checking navigation algorithms for single point positioning (SPP) in signal-degraded scenarios [7,8,9,10]. Unfortunately, with the deployment and growing availability of GNSS constellations, solution separation methods present challenging scalability issues since their computation complexity constitutes a combinatorial problem with the number of observations and outliers, eventually becoming an intractable problem.

Robust statistics provides an alternative framework for the definition of navigation methods resilient against multiple erroneous observations. Originally suggested for general data analysis in the early 1970s [11,12,13], robust estimators have experienced substantial research growth and their use has extended to manifold fields: signal processing [14,15,16], biomedical [17,18], power systems [19], etc. Within the scope of GNSS, robust methods have been successfully applied to enhance receivers with anti-jamming capabilities based on the so-called Robust Interference Mitigation [20,21,22,23,24,25,26]. The application of robust estimators to compute position, velocity, and time (PVT) solutions in satellite-based navigation has also appealed numerous authors, both for memory-less SPP [27,28,29,30] and for recursive estimation [31,32,33]. In that PVT context, the performance of robust techniques has been demonstrated on both simulated and real data, and this paper attempts to characterize those estimators in terms of quantities relevant to the robust statistics literature. This paper focuses on the SPP problem, thus purposely does not consider precise point positioning (PPP) or real-time kinematic (RTK) approaches, which typically involve more complex estimates and the application of different methodologies [34,35,36] to the ones investigated here.

This work introduces the principles of robust statistics for regression problems and presents three of the most popular robust methods: M-, S-, and MM-estimators. Besides, a comprehensive guide on the implementation of such techniques for solving the GNSS SPP problem is detailed. Moreover, the specific challenges on the application of robust estimators for GNSS positioning are discussed. Simulation experiments were carried out to evaluate the positioning capabilities of the M-, S-, and MM-estimators against classical LS. In those experiments, the pseudorange observations were contaminated with a percentage of outliers, ranging from 10% to 40 %, of different magnitude. In addition, the Gaussian efficiency and the capability of mitigating the effects of outliers is addressed over different data sizes, to verify the importance of data redundancy for the performance of robust estimators. Finally, the paper is concluded with a set of real data experiments, where standard and robust SPP solutions are compared in a vehicular scenario, which contains intervals of harsh propagation conditions that exemplify the benefits of robust SPP techniques. This paper extends [37] with additional analysis of the robust methods, the definition of the loss-of-efficiency concept for robust PVT estimation, and additional experimental discussions using real data in a vehicular scenario.

The rest of the paper is organized as follows. In Section 2, the basics of robust estimation are introduced. Section 3 relates the specific implementation details of using robust techniques in the context of GNSS single point positioning. The concept of loss-of-efficiency for PVT robust estimators is introduced in Section 4. Section 5 presents the results and discusses the performance of robust estimators in both synthetic and real experiments. Finally, Section 6 concludes the paper with an outlook and discussion of future work directions.

## 2. Robust Statistics Principles

Classical regression methods assume perfect knowledge of the probability distribution that the data obey. Particularly, parametric models are typically considered [38]. A traditional way to represent “well-behaved” data is to assume that the underlying noise is normally distributed, that is, $\eta ∼N\left(\mu ,\sigma^{2}\right)$, with known mean and variance, μ and $\sigma^{2}$. If this assumption holds, the LS estimate is known to be optimal. However, several real-world measurements have confirmed the presence of heavy-tailed (or *approximately normal*) noise [39,40,41], causing estimators derived from the Gaussian probability model to be biased or even to break down [14]. Under these circumstances, the robust estimators become relevant, given their capacity to provide *close-to-optimal* results in non-nominal conditions. The concept of approximate normality can be formalized by considering a proportion 1−ε of the observations to be effectively following a Gaussian distribution, while a complementary portion 0≤ε≤1 of the data being contaminated by an unknown (potentially) non-Gaussian distribution,

(1) $$\eta ∼\left(1-\varepsilon \right)F+\varepsilon \;H$$

where $F=N\left(\mu ,\sigma^{2}\right)$ is the nominal Gaussian distribution and *H* is an arbitrary contaminating distribution. Observations following the assumed *F* distribution are commonly referred to as *inliers*, while the corrupted observations are regarded as *outliers*. Notice that another approach for modeling outliers involves the use of *heavy-tailed* distributions, whose tails tend to zero at a slower rate than the Gaussian distribution. Cauchy, Laplace, Student-t, or α-stable distributions are examples of such heavy-tailed densities. The remainder of this section introduces basic notions in robust statistics and details some of the most well-known robust estimators for regression problems. For a detailed theoretical analysis of robust statistics, the reader is referred to classical textbooks [13,42,43], or the recent works [14,44] for its application to a variety of signal processing problems. The peculiarities of applying these methods to GNSS SPP are discussed in Section 3, as well as their validation using both synthetic and real data experiments in Section 5.

### 2.1. Dictionary of Robust Statistics Terms

In [45], Huber described the main notations of robustness in analogy to the stability of a bridge: (i) the qualitative aspect: a small perturbation should induce small effects; (ii) the breakdown aspect: how big could a perturbation be before the bridge would fall apart; and (iii) the infinitesimal aspect: how is the structure altered under the effects of infinitesimal perturbations. This section covers the basic concepts of robust statistics.

First, *qualitative robustness* is described adopting Hampel’s definition [11]. In plain words, if a bounded change in the distribution of the observations is seen as a bounded change in the distribution of the estimates, then the claim is that the estimator is robust. More precisely, let $X=\left\{x_{1},\cdots ,x_{n}\right\}$ be a set of i.i.d. observations from a distribution *F*, and let $T_{n}=T_{n}\left(X\right)$ be a sequence of estimates. Then, $T_{n}$ is called *robust* at $F=F_{0}$ if the sequence of maps of distributions ($L_{F}\left(T_{n}\right)$ stands for the distribution of an estimator (or test statistic) $T_{n}$ under *F*), $L_{F}\left(T_{n}\right)$ is equicontinuous at $F_{0}$, that is, if we take a suitable distance $d_{*}$, in the space of probability measures, and assume that for all $\delta_{2}>0$ there exists a $\delta_{1}>0$ such that,

(2) $$d_{*}\left(F_{0},F\right)\leq \delta_{1}\Rightarrow d_{*}\left(L_{F_{0}}\left(T_{n}\right),L_{F}\left(T_{n}\right)\right)\leq \delta_{2}\;.$$

Another important concept is that of *breakdown point* $\varepsilon^{*}$ of an estimator, defined as the smallest percentage of contamination that can cause the estimator to take on arbitrarily large aberrant value [11]. Later, the concept of breakdown point on finite sets was introduced in [46]. Consider any sample X of *n* observations and any estimator $T≜T_{n}$. The corrupted sample $X^{\prime }$ is obtained via ε-replacement, for which a random subset of size *m* of the original X samples is replaced by arbitrary values, with a contamination fraction of ε=m/n. The maximum estimation bias due to ε-contaminated is defined as

(3) $$MC\left(\varepsilon ;T,X\right)=\sup\left|T\left(X^{\prime }\right)-T\left(X\right)\right|$$

where the supremum is evaluated over all the set of ε-corrupted samples [46]. Thus, the breakdown point $\varepsilon^{*}$ of an estimator *T* reads:

(4) $$\varepsilon^{*}\left(T,X\right)=\inf\left\{\varepsilon :MC\left(T,X\right)=\infty \right\}$$

For an in-depth discussion on the breakdown point of the most relevant robust estimators, the reader might refer to [47]. The *influence function* IF, first introduced by Hampel [48] under the name *influence curve*, has often been considered as the most useful heuristic tool of robust statistics [42,44]. The IF measures the change of the estimator *T* at the distribution *F* when the sample contains a fraction ε of outliers, as

(5) $$IF\left(x,T(F),F\right)=\lim_{\varepsilon \to 0}\frac{T\left(\left(1-\varepsilon \right)F+\varepsilon \;\delta_{x}\right)-T\left(F\right)}{\varepsilon }$$

where *x* is the position of the infinitesimal contamination and $\delta_{x}$ is the point-mass probability at *x*. IF’s main use is to assess the relative influence of individual observations toward the value of an estimate. If it is unbounded, an outlier might cause trouble.

Robust estimators provide resiliency to outliers, but they do it at the price of some performance degradation under the nominal model, that is, when all observations are inliers. Such degradation is quantified via the so-called *loss-of-efficiency* (LoE), defined as the performance ratio between a robust estimator and the optimal method under the nominal noise model. LoE is also known as *relative* or *Gaussian efficiency*, when the underlying assumed model is Gaussian, in which case the optimal estimator is the (weighted) LS.

### 2.2. Robust Estimates for Regression Problems

Consider a linear regression problem $y_{i}=z_{i}^{⊤}x+\eta_{i}$, with i=1,⋯,n, and x the vector of unknown parameters, or in vector form, y=Zx+η with Z defined with the different $z_{i}^{⊤}$ in its rows. The noise vector η is assumed to be independent and identical along the set of observations. We can define a vector r=y−Zx of observation residuals. The regression is generally solved applying a LS estimator (minimization of the $\ell_{2}$-norm of the residuals),

(6) $${\hat{x}}_{LS}=\arg\min_{x}||y-{Zx||}^{2}\Rightarrow \arg\min_{x}\sum_{i=1}^{n}{\left(\frac{r_{i}\left(x\right)}{\sigma }\right)}^{2}\;,$$

which is optimal when the Gaussian noise assumption for η holds. However, it lacks robustness since a single (arbitrarily large) outlier could completely spoil the estimation. A first approach towards protecting against outlying measurements is the *least-absolute value* (LAV) or $\ell_{1}$, consisting on the substitution of the squared residuals as

(7) $${\hat{x}}_{\ell_{1}}=\arg\min_{x}\sum_{i=1}^{n}\left|\frac{r_{i}\left(x\right)}{\sigma }\right|\;.$$

Nonetheless, the $\ell_{1}$ method retains a sum of residuals and thus the influence of outliers is still unbounded. This problem can be generalized by considering a general loss function ρ(x) (referred to as the ρ-function), and then reformulating the regression as

(8) $$\hat{x}=\arg\min_{x}\sum_{i=1}^{n}\rho \left(\frac{r_{i}\left(x\right)}{\sigma }\right)\;.$$

**Figure 1:**
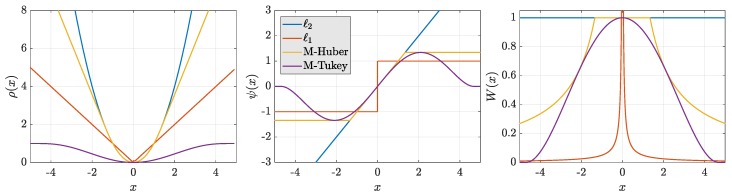
Illustration of the loss (**left**), score (**middle**) and weighting (**right**) functions for different classical and robust estimators. Here, the families of Huber and Tukey functions are depicted with parameters a=1.345 and c=4.685, respectively.

For instance, considering a scalar variable *x* (which is related to the previous definitions as $x=r_{i}\left(x\right)/\sigma$), $\rho_{\mathrm{LS}}\left(x\right)=x^{2}$ and $\rho_{\ell_{1}}\left(x\right)=\left|x\right|$ correspond to the aforementioned LS and LAV estimation approaches. The framework of robust statistics proposes loss functions $\rho \left(\cdot \right)$ such that the estimates are nearly optimal when the noise follows the assumed distribution (e.g., normal) and nearly optimal when the noise departs from it. The score function (referred to as the ψ-function) is defined as the derivative of an estimator loss function $\psi \left(x\right)=\frac{\partial \rho (x)}{\partial x}$. Several robust estimators of regression have been proposed in the literature, the most popular being: (i) M-estimate; (ii) S-estimate; and (iii) MM-estimate. In the sequel, the loss functions for robust statistics are introduced, as well as some relevant properties, for which Figure 1 provides some pictorial support.

#### 2.2.1. Huber and Tukey Families of Loss Functions

The key idea behind robust estimation is to use loss functions which appropriately penalize outliers in the measurements. Loss functions can be classified according to the shape of their score function ψ as *monotone* or *recesdending*. Among the redescending category, estimators for which $\psi \left(x_{r}\right)=0,\;x_{r}<\infty$ are denoted as *strongly redescending* [44]. Several loss functions exist in the literature, the most common being Huber and Tukey’s bisquare families of functions. The family of monotone Huber functions is defined as (again using an arbitrary scalar variable *x*)

(9) $$\begin{array}{cc} \; & \rho_{a}^{H}\left(x\right)=\left\{\begin{array}{ccc} x^{2} & \mathrm{if} & |x|\leq a\\ 2a|x|-a^{2} & \mathrm{if} & |x|>a \end{array}\right., \end{array}$$

(10) $$\begin{array}{cc} \; & \psi_{a}^{H}\left(x\right)=\left\{\begin{array}{ccc} x & \mathrm{if} & |x|\leq a\\ a\;\mathrm{sign}(x) & \mathrm{if} & |x|>a \end{array}\right., \end{array}$$

(11) $$\begin{array}{cc} \; & W_{a}^{H}\left(x\right)=\min\left\{1,\frac{a}{\left|x\right|}\right\}, \end{array}$$

then $\rho_{a}^{H}\left(x\right)$ is quadratic around 0 and increases linearly with *x*. In the case of location estimation, the limit cases, a→∞ and a→0, correspond to the mean and median estimates, respectively. The Huber loss function constitutes a combination of the $\rho_{\ell_{2}}$ and $\rho_{\ell_{1}}$ functions, behaving as a LS for small errors and as LAV for larger ones. The parameter *a* is chosen based on the target asymptotic relative efficiency (ARE) at a distribution. Thus, $a_{0.95}=1.345$ indicates that the M-estimator based on Huber’s loss function poses an ARE of 0.95 at the standard normal distribution [44].

To achieve robustness, a desirable property of ρ-functions is boundedness, which implies redescending ψ-functions that tend to 0 at infinity. A popular choice is the Tukey’s bisquare or biweight family of functions,

(12) $$\begin{array}{cc} \; & \rho_{c}^{B}\left(x\right)=\left\{\begin{array}{ccc} 1-{\left(1-{\left(\frac{x}{c}\right)}^{2}\right)}^{3} & \mathrm{if} & |x|\leq c\\ 1 & \mathrm{if} & |x|>c \end{array}\right. \end{array}$$

(13) $$\begin{array}{cc} \; & \psi_{c}^{B}\left(x\right)=x{\left(1-{\left(\frac{x}{c}\right)}^{2}\right)}^{2}I\left(|x|\leq c\right), \end{array}$$

(14) $$\begin{array}{cc} \; & W_{c}^{B}\left(x\right)={\left(1-{\left(\frac{x}{c}\right)}^{2}\right)}^{2}I\left(|x|\leq c\right), \end{array}$$

with c>0 a constant parameter and I(|x|≤c) the indicator function, i.e., I(|x|≤c)=1 if |x|≤c and 0 if |x|>c. Tukey’s loss function is nonconvex and bounded, which makes it robust to large outliers, whose influence is completely denied. Similar to Huber-based M-estimation, the value of the parameter *c* controls the degree of robustness and it is chosen to achieve certain ARE [44]. For 95% efficiency at the Gaussian distribution, Tukey parameter $c_{0.95}=4.685$.

#### 2.2.2. M-Estimator

The M-estimate of regression is defined as

(15) $${\hat{x}}_{M}=\arg\min_{x}\sum_{i=1}^{n}\rho \left(\frac{r_{i}\left(x\right)}{\hat{\sigma }}\right),$$

with $\hat{\sigma }$ an auxiliary estimate of the scale of errors, or, equivalently, as the solution to

(16) $$\sum_{i=1}^{n}\psi \left(\frac{r_{i}\left(x\right)}{\hat{\sigma }}\right)\frac{\partial \left(r_{i}\left(x\right)/\hat{\sigma }\right)}{\partial x}=0\;,$$

which is commonly solved by an Iteratively Reweighted LS (IRLS) procedure, with an instrumental weight function defined as

(17) $$W\left(x\right)=\left\{\begin{array}{c} \psi (x)/x,\;\mathrm{if}\;x\neq 0\\ \psi^{\prime }\left(0\right),\;\mathrm{if}\;x=0 \end{array}\right.,$$

to provide the convenient alternative formulation,

(18) $$\sum_{i=1}^{n}W\left(r_{i}/\hat{\sigma }\right)\;\frac{r_{i}}{\hat{\sigma }}\;\frac{\partial \left(r_{i}/\hat{\sigma }\right)}{\partial x}=0\;.$$

Solving such system requires finding the estimate as well as the weights for each of the observations according to the corresponding weighting function. Notice that a normalization using the dispersion of the residuals $\hat{\sigma }$ is included in the formulation, because these estimates are not scale equivariant. An estimate of the residuals dispersion must be used, for instance, the normalized median absolute deviation (MAD), defined as

(19) $${\hat{\sigma }}_{\mathrm{MAD}}\left(x\right)=c_{m}\;\mathrm{Med}(|x-\mathrm{Med}\left(x\right)|)$$

being Med(x) the median of x, and $c_{m}$ a normalizing constant (≈1.4815 to make MAD consistent with the usual parameter σ at Gaussian distributions) [49]. Instead of using an auxiliary scale estimate for the M-estimation, it is also possible to perform a joint regression of the vector of unknown parameters and the scale [42].

Notice as well the relevance on the choice of monotone against redescending loss functions. Monotone estimators constitute a convex optimization problem, for which the uniqueness of the solution is guaranteed and the starting point only influences the convergence rate [44]. Contrarily, redescending estimators suffer from the defect of requiring regularity conditions for their uniqueness and continuity [47]. A more extentsive discussion on this matter can be found in Section 5.1 with the pictorial support of a GNSS-related example.

#### 2.2.3. S-Estimator

The S-estimate of regression is defined as the estimator that minimizes the robust scale M-estimate,

(20) $${\hat{x}}_{S}=\arg\min_{x}s_{M}\left(r\left(x\right)\right),$$

with $s_{M}\left(r\left(x\right)\right)$ the M-estimate of scale, which satisfies

(21) $$\frac{1}{n}\sum_{i=1}^{n}\rho \left(\frac{r_{i}\left(x\right)}{s_{M}\left(r\left(x\right)\right)}\right)=b,$$

and, thus,

(22) $${\hat{x}}_{S}=\arg\min_{x}\sum_{i=1}^{n}\rho \left(\frac{r_{i}\left(x\right)}{\hat{s}}\right)\;,\;\hat{s}=s_{M}\left(r\left({\hat{x}}_{S}\right)\right)\;.$$

Again, this is solved by an IRLS approach. A typical choice for the ρ-function is the bisquare scale with $\rho \left(x\right)=\min\{1-{\left(1-x^{2}\right)}^{3},1\}$ and b=0.5. In this case, $W\left(x\right)=\min\left\{3-3x^{2}+x^{4},1/x^{2}\right\}$, where it is clear that larger values of *x* have smaller weights. S-estimator is characterized by a high breakdown point, while attaining a low efficiency at the normal distribution.

#### 2.2.4. MM-Estimator

The MM-estimator is designed to achieve both high efficiency and high breakdown point simultaneously. Consider two bounded loss functions, $\rho_{0}$ and $\rho_{1}$, which satisfy $\rho_{1}<\rho_{0}$. Then, the MM estimator is defined as

(23) $${\hat{x}}_{MM}=\arg\min_{x}\sum_{i=1}^{n}\rho_{1}\left(\frac{r_{i}\left(x\right)}{s_{M}\left(r\left({\hat{x}}_{0}\right)\right)}\right),$$

where ${\hat{x}}_{0}$ is a consistent estimator of x that has a high breakdown point, and $s_{M}\left(r\left({\hat{x}}_{0}\right)\right)$ is the M-estimate of scale of the residuals of ${\hat{x}}_{1}$, computed using $\rho_{0}$ and *b*.

The MM-estimate is implemented in three steps:

(1) Compute an initial consistent S-estimate of x, namely ${\hat{x}}_{0}$, with a high breakdown point but possibly low normal efficiency.
(2) Compute an M-estimate of the scale of the residuals $s_{M}\left(r\left({\hat{x}}_{0}\right)\right)$ using the high breakdown point estimate ${\hat{x}}_{0}$.
(3) Compute the regression M-estimate initialized at ${\hat{x}}_{0}$, considering the robust scale estimate $s_{M}\left(r\left({\hat{x}}_{0}\right)\right)$ and using a recursive IRLS approach.

## 3. Robust Statistics for GNSS Positioning

The GNSS-based positioning principle consists in solving a geometric problem from the measured ranges to the visible satellites, whose positions are known. Assuming that n≥4 satellites are tracked, then the observation model to relate the code pseudoranges to the unknown receiver coordinates is as follows:

(24) $$R_{i}={∥p_{i}-p∥}^{2}+\delta t-\delta t_{i}+I_{i}+Tr_{i}+\eta_{i}$$

where the subscript i={1,⋯,n} refers to the *i*th satellite, $R_{i}$ is the observed pseudorange, $p_{i}$ and p denote the satellite and receiver positions respectively, and δt and $\delta t_{i}$ are the clock offsets of the receiver and the satellite (in m). In addition, $I_{i}$ and $Tr_{i}$ denote the ionospheric and tropospheric corrections and $\eta_{i}$ gathers the remaining unmodeled errors (e.g., multipath effects, instrumental delays, phase biases, etc.). Solving the system of equations in Equation (24) can be formulated as a regression problem:

(25) $$y=h\left(x\right)+\eta$$

where y is the *n*-dimensional observation vector of pseudoranges, $h\left(\cdot \right)$ is the observation model from Equation (24) and $x={\left[p^{⊤},\delta t\right]}^{⊤}\in R^{p}$ is the unknown parameter vector. The dimension of the state estimate *p* depends on the number of constellations used (three for positioning plus one per each GNSS constellation used). In the context of GNSS, the LS adjustment is the most commonly used method for the estimation of the regression problem of Equation (25). Since GNSS SPP involves a nonlinear observation model, the problem is typically linearized and solved applying an iterative Gauss–Newton method as

(26) $$\begin{array}{cc} {\hat{\Delta x}}^{k} & ={\left(H^{⊤}WH\right)}^{-1}H^{⊤}W\;\left(y-h\left({\hat{x}}^{k-1}\right)\right) \end{array}$$

(27) $$\begin{array}{cc} {\hat{x}}^{k} & ={\hat{x}}^{k-1}+{\hat{\Delta x}}^{k} \end{array}$$

where H is the Jacobian matrix for the observation model, also known as geometry matrix. That linearization is performed around some guess point ${\hat{x}}^{k-1}$ for the *k*th iteration of the method, and $\hat{\Delta x}$ constitutes the update on the state estimate as in Equation (27). W is the weighting matrix for the observations. Classical SPP solutions take W as the inverse of the observations covariance matrix R. Stochastic modelling of pseudorange observations has been a recurrent topic within the GNSS community. A simplification commonly used is to assume that the observations noise is uncorrelated, zero-mean and normally distributed $\eta_{i}∼N\left(0,\sigma_{i}^{2}\right)$ [50]. Thus, the covariance matrix is given by

(28) $$R=W^{-1}=\mathrm{diag}\left(\sigma_{1}^{2},\cdots ,\sigma_{n}^{2}\right)$$

where $\sigma_{i}^{2}$ is derived from combining the uncertainty of the different error sources (satellite ephemeris and clock, ionosphere, troposphere, multipath and receiver noise), as in [51,52] or from error models dependent on the satellite elevation and/or the signal carrier-to-noise density ratio [53,54,55].

Algorithm 1 describes the IRLS process for the robust estimation of the GNSS SPP. Notice that WLS (weighted least squares) refers to the iterative Gauss–Newton described in Equations (26) and (27), and MAD is defined in Equation (19). *N* and δ denote the maximum number of iterations of the iterative Gauss–Newton method and the convergence criteria, respectively. The choice of the influence function and the scale estimate is subject on the robust estimator applied—e.g., for the M-estimator, one might use the Huber function in Equation (10) and the MAD as scale estimate.

**Algorithm 1:** IRLS procedure for robust GNSS SPP.

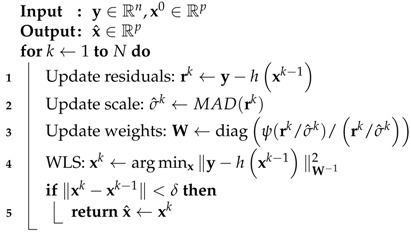



Remarkably, there are certain specific challenges associated to the GNSS-based positioning problem that we point out in this paper. First, the observation model $h\left(\cdot \right)$ is nonlinear. Thus, the IRLS procedure for finding the observations weights based on the M-estimator concatenates with the iterative LS used for dealing with the model nonlinearity. Secondly, the GNSS problem is characterized by presenting *fat* data samples, namely, there is a low redundancy of observations. Since generally only around a dozen satellites are tracked and at least four parameters are to be estimated, GNSS SPP constitutes a severe case of low redundancy regression problem [56]. Lastly, the general assumption on robust statistics of independent and identically distributed noise is not met for the GNSS case. Not only are GNSS observations noise uniquely described using stochastic models, but the assumption of independent noise can be violated for satellites of similar direction-of-arrival (e.g., for multipath and none line of sight effects), or for all satellites (e.g., under the influence of a jamming attack or an ionospheric storm).

## 4. Loss-of-Efficiency in Robust PVT Solvers

The optimal PVT solution was seen to result in a weighted least squares expression, where the weights are proportional to the inverse of the observation’s covariance matrix, as shown in Equations (26) and (27). This is optimal in the MSE sense and under perfect model assumptions. When using robust solutions, a frequently asked question is how much the robust method degrades the performance with respect to the optimal estimator when the model actually holds true. This is quantified by the so-called LoE: the ratio of performance errors between robust and optimal methods under nominal conditions. Notice that, by definition, under nominal conditions the robust estimator is suboptimal so that ratio should be in the interval (1,∞), where 1 is the ideal case where robust and optimal methods have the same performance.

In the case of PVT solvers, we can define the LoE as the ratio of mean squared error (MSE) that the optimal and robust estimators achieve. For the case of the optimal estimator (${\hat{x}}_{o}$) in Equation (26), it is easy to show that its covariance matrix is given by

(29) $$C\left({\hat{x}}_{o}\right)={\left(H^{⊤}R^{-1}H\right)}^{-1}$$

which provides the minimal MSE error

(30) $$\mathrm{MSE}\left({\hat{x}}_{o}\right)=\mathrm{Trace}\left(C({\hat{x}}_{o})\right)$$

since the optimal estimator in Equation (26) is unbiased. Then, for a suboptimal estimator $\hat{x}$, the LoE can be defined as the ratio of MSEs:

(31) $$\begin{array}{ccc} \mathrm{LoE}(\hat{x}) & = & \frac{\mathrm{MSE}(\hat{x})}{\mathrm{MSE}({\hat{x}}_{o})}=\frac{\mathrm{Trace}\left(C(\hat{x})\right)+||\mathrm{Bias}\left(\hat{x}\right){\left|\right|}^{2}}{\mathrm{Trace}\left(C({\hat{x}}_{o})\right)} \end{array}$$

with $1<\mathrm{LoE}(\hat{x})<\infty$. The terms in the numerator related to the robust estimator under study are

(32) $$\mathrm{Bias}\left(\hat{x}\right)=x-E\left(\hat{x}\right)\;\mathrm{and}\;C\left(\hat{x}\right)=E\left(\left(\hat{x}-E\left(\hat{x}\right)\right){\left(\hat{x}-E\left(\hat{x}\right)\right)}^{⊤}\right)\;,$$

which might need to be obtained through simulations if no closed form solution can be obtained.

In summary, we propose to measure the LoE of robust PVT solvers as the ratio of MSEs of that robust estimator and the optimal estimator, under nominal conditions where no outliers are present in the data.

## 5. Test and Results

This section presents results of the described robust SPP estimators. Particularly, Section 5.1 reports a set of simulated experiments to highlight certain aspects of these estimators and provide further insights on their application to GNSS SPP. Additionally, we provide results with an experimental dataset using real data recorded over harsh propagation conditions in Section 5.2.

### 5.1. Simulated Environment

The performance of robust M-, S-, and MM-estimators, as well as classical LS for GNSS positioning, was compared based on a synthetic experimentation. Two simulation scenarios were considered: (i) a single-constellation case for which n=10 satellite observations are available; and (ii) a multi-constellation case for which four constellation are assumed to provide a total of n=40 observations. In the latter, each constellation is considered to have an independent clock offset and thus the dimension of the unknown parameter vector is seven (three for the positioning and four for the clock offsets). The combination of experiments also considers variability among the fraction of contaminated measurements ε and the magnitude α of such corrupted observations, as indicated in Table 1. In total, there were 42 different experiments and the results for each of them were obtained over $10^{4}$ Monte Carlo runs. The configuration for the robust estimators was as follows: (i) Huber-based M-estimator with $a_{0.95}=1.345$; (ii) Tukey-based S-estimator with $c_{0.95}=4.685,b=0.5$; and (iii) MM-estimator combining S-estimator for initial scale and unknown parameter estimates followed by a M-estimation (with the same parameters configuration).

The simulation of the measurements was realized based on the simplified observation model in Equation (24), where atmospheric- and satellite-related effects (ionospheric, tropospheric, and ephemeris errors) were disregarded. The vector of observation errors η stacks the errors for the inlier $\eta_{in}$ and outlier $\eta_{out}$ observations, which are distributed as follows

$$\eta ={\left[\eta_{in}^{⊤},\eta_{out}^{⊤}\right]}^{⊤},\;\eta_{in}∼N\left(0,\sigma^{2}\right),\;\eta_{out}∼N\left(0,\alpha^{2}\sigma^{2}\right),$$

where the variance σ of the healthy observations is 2 m. For each Monte Carlo run, the choice of the corrupted satellites was randomly sampled.

**Figure 2:**
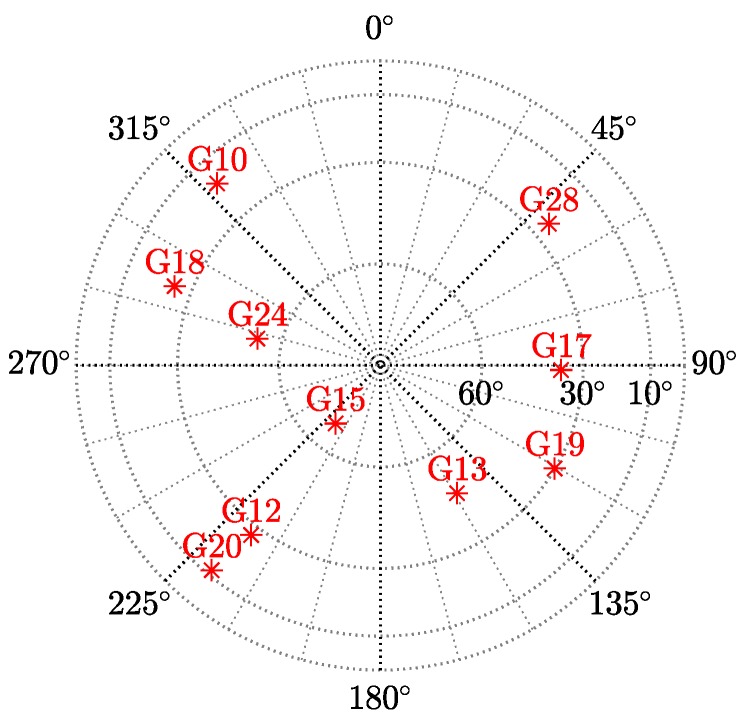
Sky plot for the single constellation simulation n=10.

For the single constellation scenario, the geometry of the satellites was based on the actual positions of GPS satellites, as shown in the sky plot of Figure 2, from a receiver located in Koblenz (Germany) in May 2017. For the multi-GNSS case, n=40 observations were simulated across four constellations (with ten satellites each). The position of the satellites were artificially “placed” on the sky according to randomly sampling the azimuth, elevation, and distance between satellites and receiver (azimuth $∼U\left(0,2\pi \right)$, elevation $∼U\left(0,\pi /4\right)$, and distance $∼N(20.200\;\left(\mathrm{km}\right),2.000\;\left({\mathrm{km}}^{2}\right))$) for each Monte Carlo instance.

**Figure 3:**
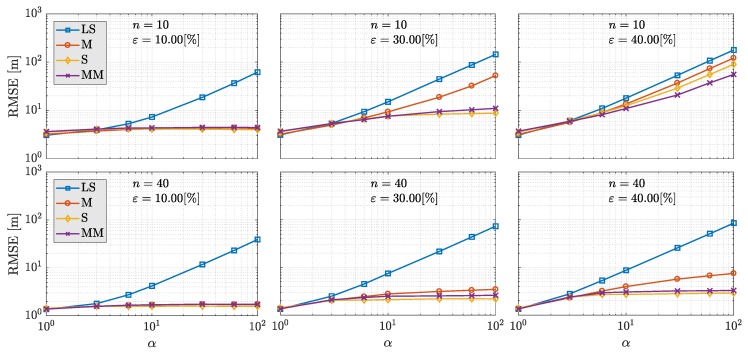
RMSE positioning error for ε∈{10,30,40}% contamination data (each column) and for n∈{10,40} (single- and multi-constellation cases, respectively) pseudorange observations (each row).

Figure 3 depicts the performance of the compared estimators, showing the positioning root mean squared error (RMSE) on the ordinate axis and the magnitude α of the outliers on the ordinate axis. The first row of Figure 3 illustrates the single constellation case, while the second row shows the multi-constellation scenario. In Figure 3, the fraction of outliers ε grows from left to right, with ε=10% (left column), ε=30% (middle column), and ε=40% (right column). A common element across all cases is, the absolute lack of robustness of the LS-estimates, whose RMSE is driven by the large errors present in the corrupted observations. Looking at the left column, M, S, and MM estimates evidence robustness at ε=10%, neglecting the effects of outliers regardless of their magnitude and number of constellations. The latter is interesting, since it appears that, even for the single constellation case (Figure 3, top left), ten observations provide enough redundancy for the estimation of four parameters and spotting a single corrupted measurement. For the middle column ε=30%, the S and MM estimators remain nearly unaffected by the outliers, indicating that their breakdown point $\varepsilon^{*}\geq 30\%$ for n≥10. On the other hand, the M estimator breaks down for the single constellation case. Finally, let us examine the right column with ε=40%. It is clear that all robust methods break down before such high fraction of contamination for the single constellation case. On the contrary, the S and MM estimators are capable of successfully bounding the effects of outliers for the multi-constellation scenario, where the large number of measurements provide with sufficient data redundancy. It becomes evident that robust methods, especially the MM estimator, represent a promising alternative to traditional ML or LS-based GNSS positioning. Especially for a near future, in which multiple GNSS constellations will be fully deployed and a large number of observations will be made available, robust methods can assure great resilience against satellite faults at a cost of minimal efficiency loss, as shown below.

**Figure 4:**
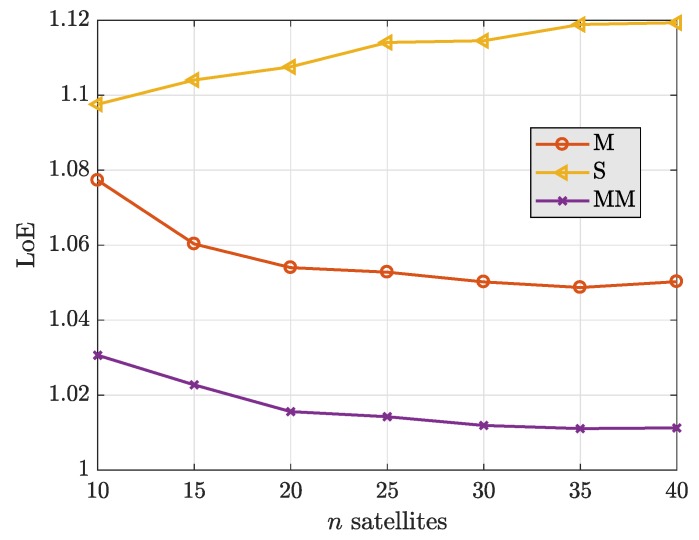
Loss-of-efficiency of the estimators as a function of the number of observations available.

The relative efficiency of the estimators with respect to the LS is studied for the nominal case—e.g., when no outlying observations are present. Figure 4 depicts the LoE of the estimators, as defined in Section 4. The S-estimator is clearly the least efficient among the evaluated methods, and its efficiency even decreases with the number of observations. Contrarily, the MM-estimator exhibits the closest performance to the LS and it can be considered as an efficient estimator. Notice that the MM efficiency scales with the number of observations, making it an appealing option for prospective multi-GNSS scenarios. Overall, one can conclude that the MM-estimator results the most interesting among the robust methods compared, offering robustness (high breakdown point) while maintaining a high efficiency at the nominal Gaussian distribution of errors.

**Figure 5:**
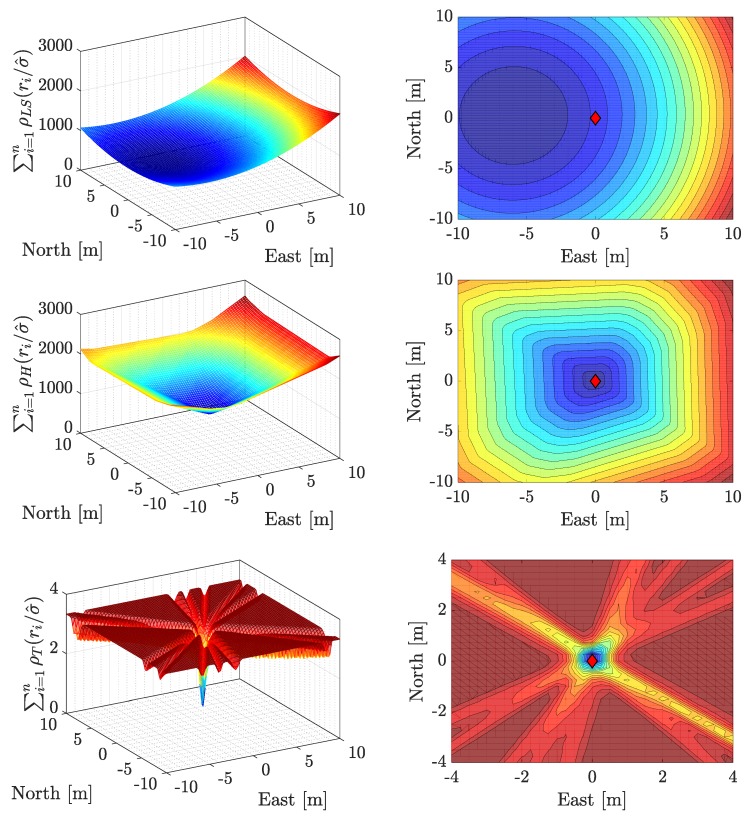
Surface (**left** column) and contour (**right** column) plot of the loss functions, projected in the east–north frame, for the LS (**top**), M-Huber (**middle**) and M-Tukey (**bottom**) estimates. The red diamond highlights the ground truth on the right column.

To gain understanding on how robust estimators actually perform for GNSS-positioning, a test scenario with a single fault was studied. Moreover, this example serves as pictorial support for the discussion on convex (based on monotone loss functions) against nonconvex (based on redescending loss functions) minimization. Making use of the single-constellation geometry of Figure 2, satellite observations are assumed to present a low variance noise ($0.1^{2}$ $m^{2}$) and a large bias inferred to satellite G17. This drives to a position error of approximately 6 m in the west direction, while the north and down directions remain mostly unaffected. Figure 5 depicts the surface (on the left column) and contour (on the right column) of the LS, M-Huber, and M-Tukey loss functions (first, second, and third rows, respectively) for the aforementioned test scenario. Besides, the ground truth solution is marked with a red diamond on the right column. Observing the first row, for the LS estimates, it becomes clear how the bias on satellite G17 is “dragging” the estimate towards the wrong direction. On the second row, the M-Huber estimate manages to discriminate the effect of the outlier and the solution becomes unbiased. Moreover, the minimization constitutes a convex problem, for which a single minimum exists and the uniqueness and stability is guaranteed. Finally, the third row shows the M-Tukey estimate. While the solution remains unaffected by the outlier, it is clear that the minimization of the nonconvex problem leads to the appearance of multiple minima. Therefore, if the initial point estimate is defect, the final estimate might not be found (due to jumps between close minima) or be spoiled (due to a local minimum).

### 5.2. Experimentation under Real Harsh Conditions

**Figure 6:**
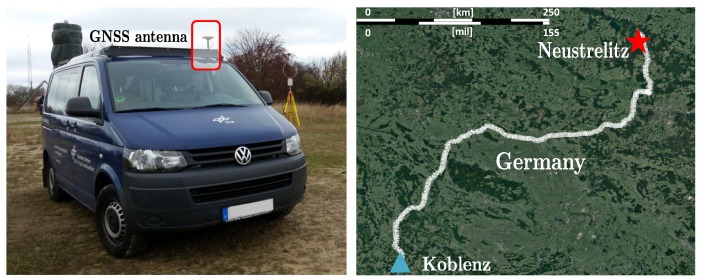
Vehicle employed for the measurement campaign (**left**). Trajectory covered during the data collection, starting in Koblenz and finishing in Neustrelitz (**right**).

To experimentally address the performance of the MM-estimator for GNSS positioning, a data collection was performed for an automotive scenario. The test vehicle was equipped with a geodetic antenna (navXperience 3G+C) connected to a geodetic GNSS receiver (Javad Delta), as shown in Figure 6 (left). The experiment was carried out on 15 May 2019 (DOY 135, UTC 10:00–18:00), covering a distance of approximately 800 km from Koblenz, in west Germany, to Neustrelitz, in northeast Germany, as illustrated in Figure 6 (right). Along the route, a wide variety of GNSS harsh conditions were confronted: urban navigation, high-speed highways, national roads under forest foliage, bridge passing, etc. Thus, the capability of the MM estimator for dealing with corrupted observations can be consistently evaluated on real multipath and NLOS conditions. The onboard GNSS receiver allows for multi-constellation (GPS, GLONASS, and Galileo), multi-frequency (L1, L2, and L5) tracking, and the sampling rate was set at 2 Hz. The ground truth reference solution was based on a dual frequency GPS+GLONASS PPP solution derived from the CSRS-PPP service [57]. Unfortunately, the PPP solution results are unavailable for the most challenging situations (e.g., tunnel or bridge crossing), thus the LS and MM performance could not be assessed during these epochs.

**Figure 7:**
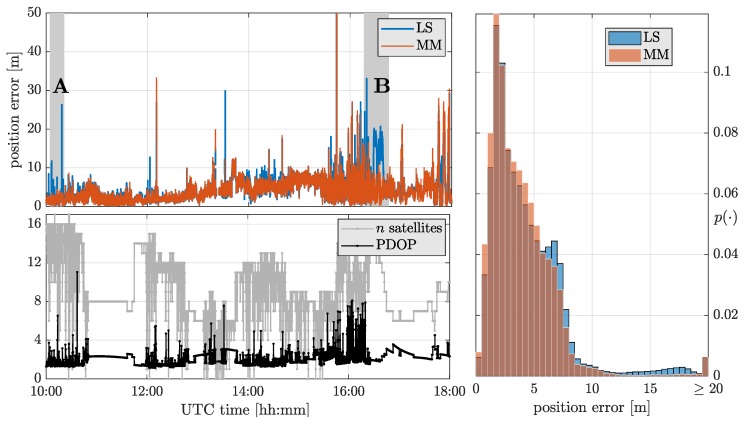
Number of GPS+Galileo satellites tracked and PDOP (**bottom left**). Squared positioning errors for the LS and MM estimators over time, and highlight on time spans A and B (**top left**). Histogram of positioning errors for LS and MM-estimator (**right**).

For the evaluation, the positioning performance of a classical LS solution was compared to the MM-estimator, which has been shown to be the most suitable among the robust methods for GNSS positioning. The estimation of the positioning solution used GPS and Galileo observations on the L1 frequency, with an elevation mask of 5, and the clock offset of Galileo was considered independent from the GPS one (hence, the number of parameters of the state estimate is of dimension five). The number of observations and the Position Dilution of Precision (PDOP) over time are illustrated in Figure 7 (bottom left). The combination of GPS and Galileo grants the availability of radio-navigation for around 96.5% of time, with often satellite tracking losses due to signal reflection and blockage.

Figure 7 (top left) depicts the three-dimensional squared positioning error over time for the LS and MM estimators. For the majority of the studied epochs—nominal opens-sky conditions for GNSS navigation—the performance of the LS and MM estimators is equivalent and the LoE of the MM is not even perceptible. This statement is supported with the results shown in Figure 7 (right), which illustrates the distribution of the positioning errors. For the bulk of the results, or errors under 5 m, LS and MM offer similar performance, with the MM even being slightly better. While LS presents a large population of positioning errors between 10 and 20 m, the MM estimator is capable of mitigating the vast majority of these errors. The largest positioning errors—for instance, shortly after 12:00 and around 15:30—cannot be mitigated by the MM estimator, which becomes as biased as the LS. This is due to a reduced satellite visibility combined with several satellites being contaminated for these epochs.

**Figure 8:**
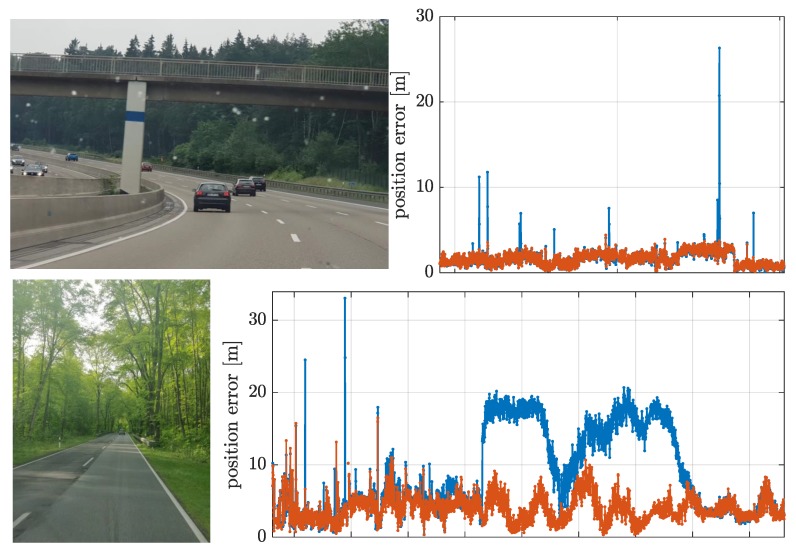
Illustration of time span “A” where multiple bridges are present (**top left**) and positioning errors during such time (**top right**). Illustration of time span “B” for navigation under dense foliage (**bottom left**) and the associated positioning errors (bottom right).

To better illustrate the outlier rejection capability of the MM estimator, time spans “A” and “B” are highlighted using a gray shaded area in Figure 7 (top left). These periods A and B of 15 and 30 min duration, respectively, are shown in detail in Figure 8, including pictures taken from the automobile during these instances. Part A corresponds to a highway where there is a succession of eight small bridges. The MM estimator results, in this case, completely unaffected by the multipath and NLOS effects. Part B corresponds to a national road surrounded by dense foliage, inducing damps on the received satellite signals. Again, the MM estimator avoids the effects of the contaminated observations, which drive the LS estimator to have errors of around 20 m.

## 6. Outlook and Future Work

This paper provides an overview of robust statistics and how it can be used to enhance the resilience of GNSS single point positioning (SPP) solutions in the presence of outliers. These large deviations from the nominal model might be caused in practice—in the GNSS context of interest here—by multipath propagation or hardware malfunctioning, for instance. SPP can be seen as a regression problem, for which this paper presents its robust alternatives leveraging the sound theory of robust statistics. At the same time, the article discusses the specific aspects of applying robust regression to GNSS SPP solvers, and supports the discussion with simulation results showing the improvements of such methods as well as their characterization. Additionally, the article considers the use of an experimental evaluation using real data, collected in a vehicular setup and including challenging propagation conditions such that the use of robust SPP methods is justified and shown in practice. Future research should provide a better (analytical) understanding of the loss-of-efficiency incurred by those methods, as well as the relaxation of the i.i.d. assumption among different satellites, and the use of robust techniques in recursive versions that yield to more sophisticated PVT solutions.

## Figures and Tables

**Table 1:** Parameters configuration for the Monte Carlo simulation.

Simulation parameters	
Number of satellites *n*	{10,40}
Percentage of outliers ε	{0,10,30,40}
Outlier magnitude α	{1,3,6,10,30,60,100}
Robust parameters	a=1.345,b=0.5,c=4.685
**Single-constellation scenario setup**	
UTC time	15/05/201709:30
Location	Koblenz, Germany
	(5021${}^{\prime }$56${}^{\prime \prime }$ N, 735${}^{\prime }$55${}^{\prime \prime }$ E)
PDOP	1.72
